# Quality of life in children and adults with epidermolysis bullosa: the QoL-REB explorative study

**DOI:** 10.1186/s13023-026-04321-6

**Published:** 2026-05-07

**Authors:** Cinzia Pilo, Laura Benedan, Valentina Morra, El Hachem M., Gianluca Tadini, Giuseppina Annicchiarico, Michela Brena, Sophie Guez, Lucia Lospalluti, Isabella L. C. Mariani Wigley, Livio Provenzi, Paolo Mariani, Serena Barello

**Affiliations:** 1 Fondazione REB, Milano, Italy; 2 Department of Economics, Management and Statistics (DEMS), University of Milano-Bicocca, Milan, Italy; 3 Dermatology Unit, Genodermatosis Research Unit, Translational Paediatrics and Clinical Genetics Research Area, Bambino Gesù Children’s Hospital, IRCCS, Rome, Italy; 4 Center for Hereditary Skin Diseases, Pediatric Dermatology Unit, University of Milan, Ospedale Maggiore Policlinico, Milan, Italy; 5 Regional Coordination of Rare Diseases (CoReMaR), Apulia Regional Agency for Health and Social Care (AReSS), Bari, Italy; 6 Pediatric Dermatology Unit, Department of Clinical Sciences and Community Health, Fondazione IRCCS Ca’ Granda Ospedale Maggiore Policlinico, Milan, Italy; 7 Pediatric-Pneumoinfectivology Unit, Fondazione IRCCS Ca’ Granda Ospedale Maggiore Policlinico, Pediatrics, Milan, Italy; 8 Dermatology Department, Azienda Universitario Ospedaliera Policlinico of Bari, Bari, Italy; 9 FinnBrain Birth Cohort Study, Turku Brain and Mind Center, University of Turku, Kiinamyllynkatu 10, Turku, 20520 Finland; 10 Centre for Population Health Research, Turku University Hospital and University of Turku, Turku, Finland; 11 Department of Psychiatry, University of Turku and Turku University Hospital, Turku, Finland; 12 Developmental Psychobiology Lab, Department of Brain and Behavioural Sciences, University of Pavia, IRCCS Mondino Foundation, Pavia, Italy; 13 WHYpsy Lab, Department of Brain and Behavioural Sciences, University of Pavia, Pavia, Italy; 14 Unit of Applied Psychology, IRCCS Mondino Foundation, Pavia, Italy

**Keywords:** Epidermolysis bullosa, Quality of life, Rare diseases, Patient-reported outcomes, Patient-centered research

## Abstract

**Background:**

Epidermolysis bullosa (EB) is a rare inherited disorder characterized by skin and mucosal fragility, with severe implications for physical, psychological, and social well-being. Research on quality of life (QoL) in EB remains limited, particularly in Italy, where systematic patient-reported outcome measures are lacking. To address this gap, Fondazione REB ETS developed a patient-centered QoL questionnaire (QoL-REB) constructed directly by patients and caregivers, with support from clinicians and researchers.

**Methods:**

We conducted a cross-sectional online survey between March and April 2024, recruiting Italian EB patients and caregivers through Fondazione REB and Debra Italia mailing lists. Participants completed the QoL-REB questionnaire, which assesses seven dimensions of QoL: physical health, autonomy, emotional well-being, family dynamics, social interactions, work/school life, and care experience. Responses were rated on a 4-point scale, with overall QoL assessed on a 0–10 scale.

**Results:**

Forty-seven individuals with EB (38 adults, 9 minors; 55% female) participated, representing multiple EB subtypes, predominantly dystrophic EB (62.4%). Mean overall QoL was rated 6/10. Pain, itching, and reduced mobility emerged as the most frequent physical challenges. Over 70% of adults reported limited autonomy in daily activities, while children experienced difficulties with walking, dressing, and sports participation. Emotional distress was common, with patients expressing concerns about future prospects, body image, and dependence on others; 43% reported a need for psychological support. Family burden was evident, with both adults and minors perceiving themselves as a strain on relatives. Social limitations, workplace and school difficulties, and dissatisfaction with healthcare services—particularly a lack of EB-specific expertise in non-reference centers—were also reported.

**Conclusions:**

This first Italian patient-led assessment highlights the pervasive and multidimensional burden of EB on QoL. Findings underscore the need for integrated, patient-centered care models that combine medical, psychological, and social support. The QoL-REB questionnaire provides a novel, comprehensive tool to capture the lived experience of EB and may serve as a framework for international adaptation and implementation.

**Supplementary Information:**

The online version contains supplementary material available at 10.1186/s13023-026-04321-6.

## Introduction

Epidermolysis bullosa (EB) is a rare, genetically inherited disorder characterised by fragility of the skin and mucous membranes, leading to recurrent blistering and a wide range of cutaneous and systemic complications [1]. Often described as “the butterfly skin disease,” EB affects not only physical health but also psychological adjustment and social participation [2, 3]. The condition is classified according to the level of cleavage within the skin into four main subtypes—EB simplex (EBS), junctional EB (JEB), dystrophic EB (DEB) and Kindler EB—each displaying different severity, clinical manifestations and care requirements [4]. Worldwide, prevalence has been estimated between 1 in 20,000 and 1 in 130,000 live births, with considerable geographical variation [5].

In Italy, a report issued by Fondazione REB ETS, a non-profit patient advocacy and support organization founded in 2017 to promote research, care and therapeutic innovation in EB, combined information from national reference centres with data from epidemiological studies [2, 6, 7] and the Orphanet database. At present, no comprehensive national registry or population-based prevalence estimates for epidermolysis bullosa are available in Italy; therefore, epidemiological figures reported in this study are derived from international literature and should be interpreted as contextual estimates rather than country-specific data. Based on these sources, prevalence is estimated at about 7 cases per million inhabitants for DEB, 18 for EBS and 1.7 for JEB, while Kindler EB remains exceptionally rare; applying an overall prevalence of 26.7 cases per million to the Italian population (~ 59 million) suggests roughly 1,570 individuals currently living with EB.

Beyond its complex clinical course, EB imposes substantial social, psychological and economic burdens on affected people and their families, making it a significant public health concern despite its rarity [8, 9]. Although physical and emotional challenges have been well documented [2, 3, 10, 11], research exploring the full multidimensional impact of EB on quality of life (QoL) remains scarce, particularly in Italy. Existing investigations have often focused on institutional or economic perspectives rather than directly capturing patients’ experiences [8], and only a few disease-specific instruments—such as the QOLEB questionnaire [12], the Scottish tool [2] and the EB-BOD questionnaire [13, 14] - are available to assess QoL in EB. While most of these tools were largely clinician-designed and primarily emphasize functional or clinical dimensions, the QOLEB questionnaire represents an important exception, as it was developed through an extensive patient-led process based on in-depth interviews and subsequent large-scale piloting [12]. Overall, relational, emotional and existential aspects remain comparatively underrepresented in many existing instruments.

Previous research has already grounded quality-of-life assessment in the perspectives of people living with epidermolysis bullosa. In addition to the QOLEB questionnaire, qualitative and validation studies from the Groningen group have further explored lived experiences and health-related quality of life through in-depth individual interviews and population-based assessment in the Dutch EB community [15]. Building on this patient-centred body of work, Fondazione REB ETS promoted and funded the development of a new instrument to assess the QoL of people with EB, designed as a complementary, multidimensional questionnaire integrating physical, functional, psycho-emotional, social and care-related domains within a single framework.

The project adopted a participatory approach in which patients and caregivers were involved throughout—from the initial qualitative exploration to the iterative refinement of items—while clinicians, psychologists and methodologists provided methodological guidance rather than directive leadership. To our knowledge, this is the first attempt to construct a QoL questionnaire for EB entirely grounded in the perspectives of those living with the condition. By engaging patients as co-creators, the QoL-REB initiative aimed to ensure that the tool would capture not only medical and functional aspects but also psychosocial, relational and existential dimensions that are often overlooked in research and practice [9, 10]. The domains identified through successive rounds of development included appearance, social participation, autonomy in daily activities, opportunities in work or school, and the emotional burden of progressive physical limitations. Given the universal relevance of these themes, the questionnaire was conceived to be adaptable for use in other cultural contexts. This patient-led strategy aligns with contemporary standards for patient-reported outcome development, such as the FDA Guidance on PRO instruments and the PCORI methodology standards, which emphasise the value of involving patients in defining outcomes that matter to them.

Building on this groundwork, the present study is, as far as we know, the first to apply the QoL-REB questionnaire within the Italian EB community. Conducted under the QoL-REB-I Project, it brought together clinicians from several reference centres, researchers with expertise in outcome measurement, and the same network of patients and caregivers who had contributed to the questionnaire’s design. The study set out to provide preliminary insights into how EB affects the everyday lives of Italian children and adults, describing both shared and age-specific challenges, with the ultimate aim of informing more patient-centred care strategies and supporting health and social policies that can improve the quality of life of people living with this rare and debilitating disorder.

## Methods

A literature review was undertaken using PubMed and Google Scholar to identify quality-of-life instruments developed for epidermolysis bullosa (EB) or other rare dermatological conditions over the past two decades, with attention to their strengths and limitations. Building on these findings, a multidisciplinary panel was convened to design a patient-centred questionnaire for assessing quality of life in EB. The panel was composed of four adults with EB, two mothers of children with EB, the president of Fondazione REB ETS, two dermatologists with expertise in EB, a psychologist, a biostatistician, and a specialist in Delphi methodology. A modified Delphi process [16] was used to achieve consensus on questionnaire content. The method followed the standard steps of expert input, anonymity to minimise social pressure, statistical aggregation of responses, and iteration with controlled feedback. Three rounds were conducted until stability of ratings was achieved, even when full consensus was not required.

Patients and caregivers first identified key concerns through an open-ended questionnaire, which was analysed using content analysis to generate preliminary domains. Successive rounds of review and refinement led to the definition of seven core areas covering physical health, autonomy, emotional well-being, family dynamics, social relationships, economic and work/school life, and experience of care. Across four iterations, 93 draft statements were examined: 54 were rephrased, eight discarded, and 15 added. The final instrument consisted of 87 items, formulated to ensure clarity and relevance for both adult and paediatric respondents. Validation focused on content validity and reliability; the full development process is reported elsewhere [17, 18]. Exploratory or confirmatory factor analysis was not performed, as the questionnaire was developed through an integrative, patient-informed process aimed at preserving content relevance and experiential salience across predefined domains. Moreover, the available sample size was intended for feasibility and reliability testing rather than for latent structure modelling.

A cross-sectional survey was then conducted to administer the questionnaire to Italian patients with EB. Data were collected between March and April 2024 using an anonymous online form created in Qualtrics and distributed via computer-assisted web interviewing (CAWI). Based on self-reported information and administration logs, participants required on average approximately 20–25 min to complete the questionnaire (median 22 min). This completion time was generally considered acceptable and did not generate reports of excessive respondent burden. Participants were recruited through the mailing lists of Fondazione REB and Debra Italia; only individuals (or caregivers of minors) who had previously provided consent to be contacted were invited. For participants under 18 years, parents or legal guardians completed the survey on their behalf. Eligibility required a confirmed diagnosis of EB and fluency in Italian. The study protocol was approved by the Comitato Etico Territoriale Lombardia 1 (protocol QoL-REB v.3_2023).

## Results

Of the 96 individuals invited to participate in the study, 47 completed the survey, corresponding to a response rate of 49%. The sample comprised 38 adults and nine minors, with a slight female predominance (55%). All major EB subtypes were represented, although dystrophic EB was the most frequent (62.4%). For participants younger than 18 years, questionnaires were completed by parents or legal guardians.

Overall, respondents rated their QoL at an average of 6 out of 10, suggesting substantial difficulties in everyday functioning. Nearly half of the participants (43%) indicated a current need for psychological support. Only 23% expressed confidence that their reference healthcare centre possessed sufficient expertise regarding EB and its available treatments, highlighting gaps in specialist knowledge and resources.

Exploration of the seven domains of the QoL-REB questionnaire revealed a multidimensional burden. Physical health problems were prominent: pain, weakness and pruritus were common, particularly during warmer months, and both adults and children reported reduced hand mobility. Functional autonomy was also severely affected. Many adults described difficulties with personal hygiene, preparing meals and managing domestic tasks, with over 70% reporting limited independence at home. Children frequently encountered obstacles in walking, dressing alone and engaging in sports, with two-thirds indicating restrictions in these activities.

Psychological and emotional aspects further shaped participants’ experiences. Adults voiced concerns about their future, body image and the risk of loneliness, while children described distress linked to dependence on others and fear of being left alone. Family life was similarly impacted: adults worried that their condition might diminish their relatives’ happiness and reported feelings of guilt, whereas children sometimes felt misunderstood or burdensome.

Social participation emerged as a sensitive area. Adults often felt uncomfortable in social contexts and faced limitations in forming or maintaining friendships and romantic relationships. Children also reported difficulties in establishing peer connections and frequently refrained from sharing information about their condition in order to avoid upsetting classmates. Economic and educational barriers were noted across the sample: adults mentioned the financial weight of EB and logistical problems at work, while younger respondents described school environments that were often unprepared to meet their needs. Finally, experience of care strongly influenced QoL. Participants valued access to professionals knowledgeable about EB and to appropriate medications, and children underlined the importance of privacy during medical procedures.

**Fig. 1:**
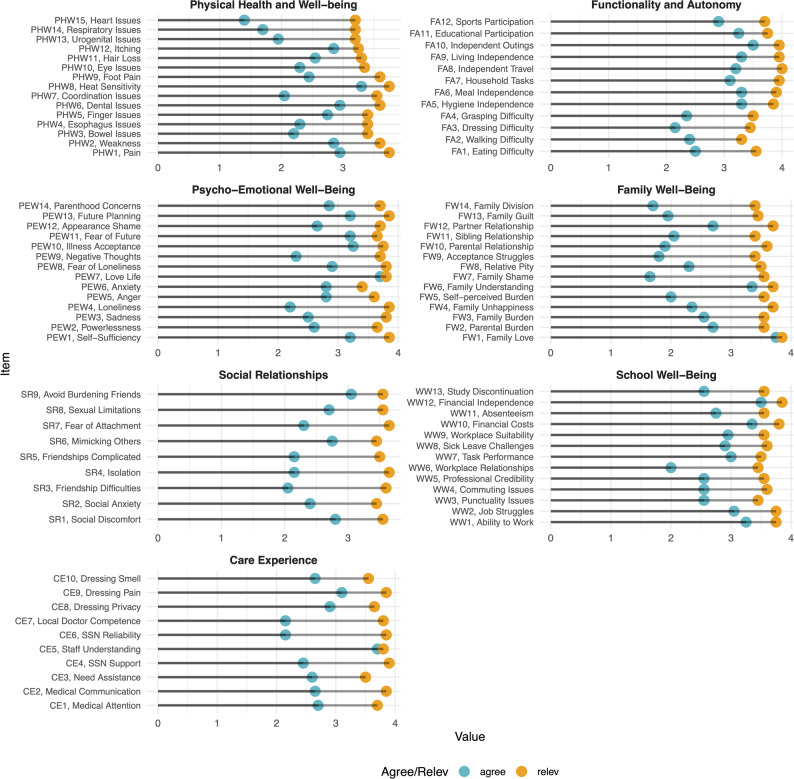
Quality of life questionnaire items response. Agree and relevance scores are reported for adults

**Fig. 2:**
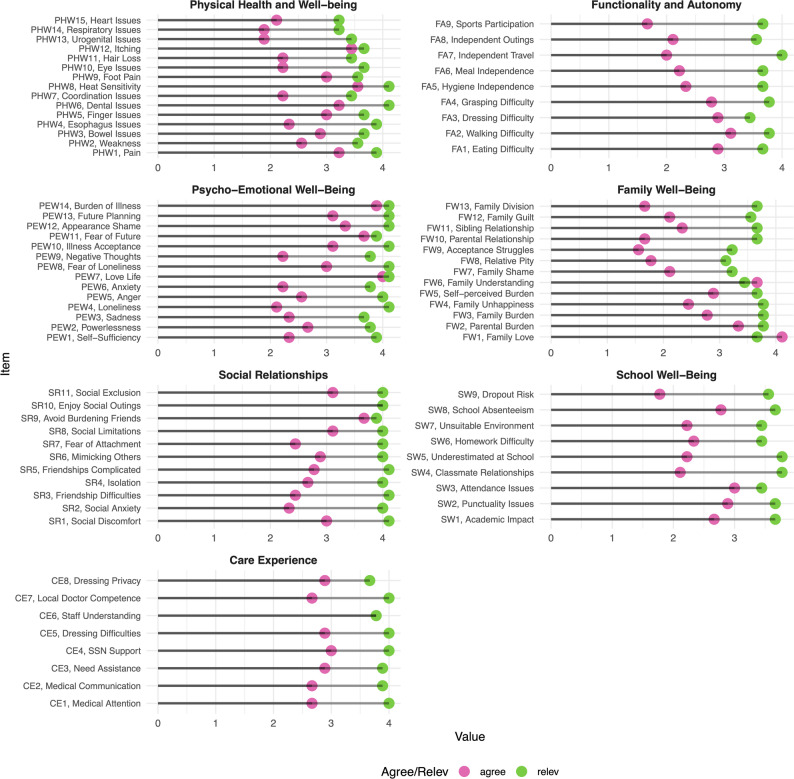
Quality of life questionnaire items response. Agree and relevance scores are reported for children

The detailed distribution of responses for each item of the adult and paediatric versions of the QoL-REB questionnaire is reported in the Supplementary Material, which provides complete wording and scoring of the instruments. Figures 1 and 2, summarize agreement and relevance scores for items across the seven domains for adult and paediatric data respectively.

## Discussion

This study offers new insights into the complex impact of EB on the QoL of Italian children and adults, using, for the first time to our knowledge, a patient-generated instrument to capture their experiences. The findings highlight the pervasive physical, psychological, social, and economic challenges faced by individuals with EB and their families, emphasising the importance of comprehensive, patient-centred models of care.

Physical symptoms remain one of the most debilitating aspects of EB. Participants frequently reported pain, itching, muscle weakness and restricted mobility, with symptoms often worsening during warmer months. These observations are consistent with previous literature on the chronic nature of EB-related discomfort and on seasonal variations in skin fragility [15, 19–23]. Advances in wound care — including modern dressings, the first topical gene therapy for DEB [24, 25], and emerging nanotechnology-based approaches — may help to accelerate healing and reduce pain [20]. Personalised pain management, combining pharmacological strategies with non-pharmacological interventions such as cognitive–behavioural therapy and mindfulness, can further enhance symptom control [26, 27]. Although assistive technologies (e.g., mobility aids, adaptive utensils) could in principle improve autonomy, the intrinsic fragility of EB skin limits the suitability of many devices; support is therefore often partial, and careful individual tailoring is essential.

Psychological distress was a prominent theme, with many participants describing anxiety, depressive feelings and social withdrawal. These results echo studies showing that visible symptoms and the stigma surrounding chronic skin conditions can significantly affect self-image and social confidence [28]. The unpredictable course of EB and the need for lifelong interventions may further compound emotional strain. Systematic inclusion of mental health professionals in EB teams, routine screening for distress, and the availability of psychotherapeutic support could help patients and caregivers to develop effective coping strategies [29]. Peer-support groups have also proven beneficial in reducing isolation and fostering resilience [2].

The burden of EB extends to the entire family. Feelings of guilt and perceptions of being a burden, together with caregiver exhaustion, were common in our sample and have been noted elsewhere [21, 30, 31]. Family-centred approaches, including counselling and respite services, can sustain caregivers’ wellbeing and improve intra-family communication. The publication of the EB-BoD questionnaire [13] and its subsequent validation in different countries, including Italy and Saudi Arabia [14, 32] shows the increasing recognition of the psychosocial load of EB on relatives. Qualitative studies also document the social consequences of EB for families [33], further supporting the need for structured support systems.

Loss of functional independence was another salient issue. Adults frequently reported obstacles in activities such as bathing, meal preparation and household tasks, while children struggled with walking, self-dressing and sports. These results mirror reports from other chronic disorders involving high physical dependency [15, 19, 34]. Rehabilitation programmes designed specifically for EB, together with careful use of protective equipment and adaptive utensils, may enhance self-efficacy and psychological wellbeing.

Economic and organisational aspects were also central. Many respondents described the heavy financial burden of treatments, dressings and travel to reference centres, in line with earlier findings on the cost of EB care [20]. Dissatisfaction with the competence of some healthcare providers emerged, indicating the need for broader professional education beyond reference centres. Training should include evidence-based wound management, pain control, psychological support and patient-centred communication [2]. Recent initiatives, such as the Italian Delphi consensus on multidisciplinary care for recessive dystrophic EB [14] and the European Reference Network position statement on EB management [35], provide valuable guidance for structuring such programmes.

Our findings also suggest that the burden of EB is gaining increasing attention worldwide, with new instruments being developed to assess both patients’ and caregivers’ perspectives, including the EB-BoD questionnaire and its cross-cultural validations [32], as well as our own QoL-REB tool. These initiatives reflect a welcome shift toward systematically addressing psychosocial dimensions alongside medical outcomes.

The present study has some limitations. The number of paediatric participants was relatively small, limiting the generalisability of results in that subgroup. The sample was restricted to Italian respondents, so cultural and organisational differences may affect applicability elsewhere. Although the questionnaire was intentionally comprehensive to capture all aspects reported by patients, its length may limit feasibility in busy clinical settings. Further studies with larger, more diverse cohorts, and the development of shorter forms for routine practice, are warranted. We nevertheless recognise these constraints as limitations and have explicitly acknowledged them in the revised manuscript, while encouraging future studies to replicate and extend the validation in larger samples. Future research may explore the development and validation of age-appropriate adaptations for children and adolescents, with wording, response formats and administration procedures tailored to different developmental stages and ethical requirements for research involving minors. Longitudinal research would help clarify how QoL evolves over time and assess the impact of integrated interventions.

## Conclusion

As far as we know, this is the first study to apply a fully patient-generated quality-of-life questionnaire to Italian people living with epidermolysis bullosa. The results show that EB affects every domain of daily life, from pain and mobility to emotions, family relationships, education, work, and access to competent care. Improving QoL for this population requires coordinated, multidimensional strategies: optimised wound and pain management, timely psychological support, family-oriented services, rehabilitation programmes, and financial and organisational measures that reduce inequities.

Education of healthcare providers and dissemination of consensus guidelines are also crucial, together with public awareness campaigns that reduce stigma and foster inclusion. The QoL-REB questionnaire, by reflecting the priorities of patients and caregivers, can serve as a practical framework for clinical assessment, shared decision-making and policy planning, ultimately contributing to better health and social outcomes for people living with this rare and demanding condition.

## Supplementary Information

Below is the link to the electronic supplementary material.

Supplementary Material 1

## Data Availability

The datasets generated and analyzed during the current study contain sensitive or potentially identifiable information and are therefore not publicly available due to ethical restrictions. Data are available from the corresponding author upon reasonable request.

## Abbreviations

EB: Epidermolysis bullosa
QoL: Quality of life
