# Correction to: Quality of life in children and adults with epidermolysis bullosa: the QoL-REB explorative study

**DOI:** 10.1186/s13023-026-04529-6

**Published:** 2026-08-07

**Authors:** Cinzia Pilo, Laura Benedan, Valentina Morra, El Hashem M., Gianluca Tadini, Giuseppina Annicchiarico, Michela Brena, Sophie Guez, Lucia Lospalluti, Isabella L. C. Mariani Wigley, Livio Provenzi, Paolo Mariani, Serena Barello

**Affiliations:** 1 Fondazione REB, Milano, Italy; 2 Department of Economics, Management and Statistics (DEMS), University of Milano- Bicocca, Milan, Italy; 3 Dermatology Unit, Genodermatosis Research Unit, Translational Paediatrics and Clinical Genetics Research Area, Bambino Gesù Children’s Hospital, IRCCS, Rome, Italy; 4 Center for Hereditary Skin Diseases, Pediatric Dermatology Unit, University of Milan, Ospedale Maggiore Policlinico, Milan, Italy; 5 Regional Coordination of Rare Diseases (CoReMaR), Apulia Regional Agency for Health and Social Care (AReSS), Bari, Italy; 6 Pediatric Dermatology Unit, Department of Clinical Sciences and Community Health, Fondazione IRCCS Ca’ Granda Ospedale Maggiore Policlinico, Milan, Italy; 7 Pediatric-Pneumoinfectivology Unit, Fondazione IRCCS Ca’ Granda Ospedale Maggiore Policlinico, Pediatrics, Milan, Italy; 8 Dermatology Department, Azienda Universitario Ospedaliera Policlinico of Bari, Bari, Italy; 9 FinnBrain Birth Cohort Study, Turku Brain and Mind Center, University of Turku, Kiinamyllynkatu 10, Turku, 20520 Finland; 10 Centre for Population Health Research, Turku University Hospital and University of Turku, Turku, Finland; 11 Department of Psychiatry, University of Turku and Turku University Hospital, Turku, Finland; 12 Developmental Psychobiology Lab, Department of Brain and Behavioural Sciences, University of Pavia, IRCCS Mondino Foundation, Pavia, Italy; 13 WHYpsy Lab, Department of Brain and Behavioural Sciences, University of Pavia, Pavia, Italy; 14 Unit of Applied Psychology, IRCCS Mondino Foundation, Pavia, Italy

**Correction to: Orphanet Journal of Rare Diseases (2026) 21:181**

10.1186/s13023-026-04321-6

Following publication of the original article [1], we have been notified that one of the authors’ first and last names were switched and, thus, tagged incorrectly.

It is now:

May El – first name

Hachem – last name

It should be:

El Hashem – first name

M. – last names

The original article was updated.
