# Supplementary material for: Quality of life in children and adults with epidermolysis bullosa: the QoL-REB explorative study
Source: Orphanet J Rare Dis. 2026 May 7;21:181. doi: 10.1186/s13023-026-04321-6 (PMC13151378; doi:10.1186/s13023-026-04321-6)
Supplement: Supplementary file 1 — Supplementary Material 1 [file 13023_2026_4321_MOESM1_ESM.docx]

**Quality of life in children and adults with epidermolysis bullosa:**

**The Qol-REB explorative study**

Cured by: Isabella Mariani Wigley

***Supplementary Material***

In this appendix, we present the QoL-REB questionnaire designed to assess the quality of life among youths and adults with epidermolysis bullosa. This scale aims to provide insights into various aspects of well-being, highlighting the experiences and challenges faced by individuals living with this condition. In both cases, subjects can rate the degree of agreement and relevance using a 4-point Likert scale.

**SECTION 1 - Children Survey**

Scale 1: Physical Health and Well−being

1. I experience pain *(PHW1, Pain)*
2. I am weak *(PHW2, Weakness)*
3. I have problems with constipation or difficulty with bowel movements *(PHW3, Bowel Issues)*
4. I have problems with my esophagus *(PHW4, Esophagus Issues)*
5. I have problems with my fingers *(PHW5, Finger Issues)*
6. I have problems with my teeth and mouth *(PHW6, Dental Issues)*
7. I have problems with motor coordination *(PHW7, Coordination)*
8. My skin worsens with heat *(PHW8, Heat Sensitivity)*
9. I have pain in my feet and cannot walk *(PHW9, Foot Pain)*
10. I have problems with my eyes *(PHW10, Eye Issues)*
11. I am losing my hair *(PHW11, Hair Loss)*
12. I have itching *(PHW12, Itching)*
13. I have problems with my urogenital system *(PHW13, Urogenital Issues)*
14. I have respiratory problems *(PHW14, Respiratory Issues)*
15. I have heart problems *(PHW15, Heart Issues)*

Scale 2: Functionality and Autonomy

1. I have difficulty eating *(FA1, Eating Difficulty)*
2. I have difficulty walking *(FA2, Walking Difficulty)*
3. I have difficulty dressing myself *(FA3, Dressing Difficulty)*
4. I have difficulty grasping things and using common objects *(FA4, Grasping Difficulty)*
5. I can manage my personal hygiene by myself *(FA5, Hygiene Independence)*
6. I can manage meals by myself *(FA6, Meal Independence)*
7. I can travel without needing help *(FA7, Independent Travel)*
8. I can go out by myself *(FA8, Independent Outings)*
9. I can engage in sports activities *(FA9, Sports Participation)*

Scale 3: Psycho−emotional Well−being

1. I feel self-sufficient *(PEW1, Self-Sufficiency)*
2. I feel powerless, demoralized, frustrated *(PEW2, Powerlessness)*
3. I feel sad *(PEW3, Sadness)*
4. I feel alone *(PEW4, Loneliness)*
5. I feel anger *(PEW5, Anger)*
6. I feel anxiety *(PEW6, Anxiety)*
7. I love life *(PEW7, Love Life)*
8. I am afraid of being alone *(PEW8, Fear of Loneliness)*
9. I have negative thoughts *(PEW9, Negative Thoughts)*
10. I have accepted my illness *(PEW10, Illness Acceptance)*
11. I am afraid of the future that awaits me *(PEW11, Fear of Future)*
12. I feel ashamed of my appearance *(PEW12, Appearance Shame)*
13. I am able to plan my future *(PEW13, Future Planning)*
14. It is heavy to live with this illness *(PEW14, Burden of Illness)*

Scale 4: Family Well−being

1. I think my family loves me *(FW1, Family Love)*
2. I think my parents are forced to always be by my side *(FW2, Parental Burden)*
3. I think it is burdensome for my family to take care of me *(FW3, Family Burden)*
4. I think that because of my illness, my family is not happy *(FW4, Family Unhappiness)*
5. I feel like a burden to my family *(FW5, Self-perceived Burden)*
6. I feel understood by my family *(FW6, Family Understanding)*
7. I think my family is ashamed of me *(FW7, Family Shame)*
8. I think I am pitiful to some relatives *(FW8, Relative Pity)*
9. I think my family struggles to accept my illness *(FW9, Acceptance Struggles)*
10. I have a difficult relationship with my parents *(FW10, Parental Relationship)*
11. I think the relationship with my siblings is or could be difficult *(FW11, Sibling Relationship)*
12. One or more family members make me feel guilty *(FW12, Family Guilt)*
13. I think my family could be divided because of me *(FW13, Family Division)*

Scale 5: Social Relationships

1. People make me feel uncomfortable (they stare at me, make comments, etc.) *(SR1, Social Discomfort)*
2. I feel anxious when I am with other people *(SR2, Social Anxiety)*
3. I struggle to make friends *(SR3, Friendship Difficulties)*
4. I give up going out with friends and/or tend to isolate myself *(SR4, Isolation)*
5. My relationship with my friends is difficult/complicated *(SR5, Friendships Complicated)*
6. When I am in company, I try to do what others are doing *(SR6, Mimicking Others)*
7. I am afraid of falling in love or becoming attached to people *(SR7, Fear of Attachment)*
8. The illness limits my social life *(SR8, Social Limitations)*
9. I try not to burden my friends with my situation *(SR9, Avoid Burdening Friends)*
10. Going out with friends makes me feel good *(SR10, Enjoy Social Outings)*
11. People exclude me because of my illness *(SR11, Social Exclusion)*

Scale 6: School Well−being

1. My school performance is affected by the illness *(SW1, Academic Impact)*
2. I have difficulty being punctual for school *(SW2, Punctuality Issues)*
3. I have difficulty getting to school *(SW3, Attendance Issues)*
4. I have difficulty relating to my classmates *(SW4, Classmate Relationships)*
5. At school, I feel underestimated because of my illness *(SW5, Underestimated at School)*
6. I have difficulty doing homework and studying *(SW6, Homework Difficulty)*
7. I have difficulties at school because the environment is not suitable for my needs *(SW7, Unsuitable Environment)*
8. I miss many days of school *(SW8, School Absenteeism)*
9. I do not think I will continue my studies *(SW9, Dropout Risk)*

Scale 7: Care Experience

1. I think doctors give me the right attention *(CE1, Medical Attention)*
2. Doctors know how to answer my questions *(CE2, Medical Communication)*
3. I would need more medical and nursing assistance *(CE3, Need Assistance)*
4. The National Health Service (SSN) provides me with everything I need for dressings *(CE4, SSN Support)*
5. Dressings are a problem *(CE5, Dressing Difficulties)*
6. I wish the medical/paramedical staff had a better understanding of the condition *(CE6, Staff Understanding)*
7. The doctors in my city know how to manage my illness *(CE7, Local Doctor Competence)*
8. When I am in the hospital, I have enough privacy to perform my dressings *(CE8, Dressing Privacy)*

**SECTION 2 - Adults Survey**

Scale 1: Physical Health and Well−being

1. I experience pain (PHW1, Pain)
2. I am weak (PHW2, Weakness)
3. I have problems with constipation or difficulty with bowel movements (PHW3, Bowel Issues)
4. I have problems with my esophagus (PHW4, Esophagus Issues)
5. I have problems with my fingers (PHW5, Finger Issues)
6. I have problems with my teeth and mouth (PHW6, Dental Issues)
7. I have problems with motor coordination (PHW7, Coordination Issues)
8. My skin worsens with heat (PHW8, Heat Sensitivity)
9. I have pain in my feet and cannot walk (PHW9, Foot Pain)
10. I have problems with my eyes (PHW10, Eye Issues)
11. I am losing my hair (PHW11, Hair Loss)
12. I have itching (PHW12, Itching)
13. I have problems with my urogenital system (PHW13, Urogenital Issues)
14. I have respiratory problems (PHW14, Respiratory Issues)
15. I have heart problems (PHW15, Heart Issues)

Scale 2: Functionality and Autonomy

1. I have difficulty eating (FA1, Eating Difficulty)
2. I have difficulty walking (FA2, Walking Difficulty)
3. I have difficulty dressing myself (FA3, Dressing Difficulty)
4. I have difficulty grasping things and using common objects (FA4, Grasping Difficulty)
5. I can manage my personal hygiene by myself (FA5, Hygiene Independence)
6. I can manage meals by myself (FA6, Meal Independence)
7. I can do cleaning and household chores (FA7, Household Tasks)
8. I can travel without needing help (FA8, Independent Travel)
9. I can live alone (FA9, Living Independence)
10. I can go out by myself (FA10, Independent Outings)
11. I can participate in educational activities (such as school/university) (FA11, Educational Participation)
12. I can engage in sports activities (FA12, Sports Participation)

Scale 3: Psycho−emotional Well−being

1. I feel self-sufficient (PEW1, Self-Sufficiency)
2. I feel powerless, demoralized, frustrated (PEW2, Powerlessness)
3. I feel sad (PEW3, Sadness)
4. I feel alone (PEW4, Loneliness)
5. I feel anger (PEW5, Anger)
6. I feel anxiety (PEW6, Anxiety)
7. I love life (PEW7, Love Life)
8. I am afraid of being alone (PEW8, Fear of Loneliness)
9. I have negative thoughts (PEW9, Negative Thoughts)
10. I have accepted my illness (PEW10, Illness Acceptance)
11. I am afraid of the future that awaits me (PEW11, Fear of Future)
12. I feel ashamed of my appearance (PEW12, Appearance Shame)
13. I am able to plan my future (PEW13, Future Planning)
14. I worry about being able to have children (PEW14, Parenthood Concerns)

Scale 4: Family Well−being

1. I think my family loves me (FW1, Family Love)
2. I think my parents are forced to always be by my side (FW2, Parental Burden)
3. I think it is burdensome for my family to take care of me (FW3, Family Burden)
4. I think that because of my illness, my family is not happy (FW4, Family Unhappiness)
5. I feel like a burden to my family (FW5, Self-perceived Burden)
6. I feel understood by my family (FW6, Family Understanding)
7. I think my family is ashamed of me (FW7, Family Shame)
8. I think I am pitiful to some relatives (FW8, Relative Pity)
9. I think my family struggles to accept my illness (FW9, Acceptance Struggles)
10. I have a difficult relationship with my parents (FW10, Parental Relationship)
11. I think the relationship with my siblings is or could be difficult (FW11, Sibling Relationship)
12. I think the relationship with my partner is or could be difficult (FW12, Partner Relationship)
13. One or more family members make me feel guilty (FW13, Family Guilt)
14. I think my family could be divided because of me (FW14, Family Division)

Scale 5: Social Relationships

1. People make me feel uncomfortable (they stare at me, make comments, etc.) (SR1, Social Discomfort)
2. I feel anxious when I am with other people (SR2, Social Anxiety)
3. I struggle to make friends (SR3, Friendship Difficulties)
4. I give up going out with friends and/or tend to isolate myself (SR4, Isolation)
5. My relationship with my friends is difficult/complicated (SR5, Friendships Complicated)
6. When I am in company, I try to do what others are doing (SR6, Mimicking Others)
7. I am afraid of falling in love or becoming attached to people (SR7, Fear of Attachment)
8. The illness limits my sexual activity (SR8, Sexual Limitations)
9. I try not to burden my friends with my situation (SR9, Avoid Burdening Friends)

Scale 6: Work Well−being

1. I can work (WW1, Ability to Work)
2. I struggle to find/keep a job (WW2, Job Struggles)
3. I think I might have/have difficulties being punctual at work (WW3, Punctuality Issues)
4. I think I might have/have difficulties getting to work (WW4, Commuting Issues)
5. I think I might have/have difficulties gaining professional credibility with colleagues and/or clients (WW5, Professional Credibility)
6. I think I might have/have difficulties relating to others in a work environment (WW6, Workplace Relationships)
7. I think I could/can perform all tasks (WW7, Task Performance)
8. I think I might have/have difficulties obtaining sick leave (WW8, Sick Leave Challenges)
9. I think I might have/have difficulties at my workplace as it is not suitable for my needs (WW9, Workplace Suitability)
10. I incur many expenses (WW10, Financial Costs)
11. I miss many days of study/work (WW11, Absenteeism)
12. I am financially self-sufficient (WW12, Financial Independence)
13. I was unable to continue my studies (WW13, Study Discontinuation)

Scale 7: Care Experience

1. I think doctors give me the right attention (CE1, Medical Attention)
2. Doctors know how to answer my questions (CE2, Medical Communication)
3. I would need more medical and nursing assistance (CE3, Need Assistance)
4. The National Health Service (SSN) provides me with all the supplies and medications prescribed by my referral center (CE4, SSN Support)
5. I wish the medical/paramedical staff had a better understanding of the condition (CE5, Staff Understanding)
6. I feel I can rely on the National Health Service (SSN) for my illness (CE6, SSN Reliability)
7. The doctors in my city know how to manage my illness (CE7, Local Doctor Competence)
8. When I am in the hospital, I have enough privacy to perform my dressings (CE8, Dressing Privacy)
9. I experience pain during dressing changes (CE9, Dressing Pain)
10. The smell of the ulcers and/or dressings is unpleasant (CE10, Dressing Smell)
